# Identification and biochemical characterization of the fructokinase gene family in *Arabidopsis thaliana*

**DOI:** 10.1186/s12870-017-1031-5

**Published:** 2017-04-26

**Authors:** John W. Riggs, Philip C. Cavales, Sonia M. Chapiro, Judy Callis

**Affiliations:** 1 Department of Molecular and Cellular Biology, College of Biological Sciences, University of California, 1 Shields Ave, Davis, CA 95616 USA; 2 Present Address: Department of Plant and Microbial Biology, University of California, Berkeley, CA 94720 USA; 3 Present Address: Joint Bioenergy Institute, Emeryville, CA 94608 USA

**Keywords:** Carbohydrate metabolism, Fructokinase, Carbohydrate kinase, Fructose, Enzyme, Arabidopsis, metabolism, pfkB

## Abstract

**Background:**

Fructose is an abundant sugar in plants as it is a breakdown product of both major sucrose-cleaving enzymes. To enter metabolism, fructose is phosphorylated by a fructokinase (FRK). Known FRKs are members of a diverse family of carbohydrate/purine kinases known as the phosphofructokinase B (pfkB) family. The complete complement of active fructokinases has not been reported for any plant species.

**Results:**

Protein sequence analysis of the 22 *Arabidopsis thaliana* pfkB members identified eight highly related predicted proteins, including one with previously demonstrated FRK activity. For one, At1g50390, the predicted open reading frame is half the size of active FRKs, and only incompletely spliced RNAs were identified, which led to a premature stop codon, both indicating that this gene does not produce active FRK. The remaining seven proteins were expressed in *E. coli* and phosphorylated fructose specifically in vitro leading us to propose a unifying nomenclature (FRK1–7). Substrate inhibition was observed for fructose in all FRKs except FRK1. Fructose binding was on the same order of magnitude for FRK1–6, between 260 and 480 μM. FRK7 was an outlier with a fructose Km of 12 μM. ATP binding was similar for all FRKs and ranged between 52 and 280 μM. YFP-tagged AtFRKs were cytosolic, except plastidic FRK3. T-DNA alleles with non-detectable wild-type RNAs in five of the seven active FRK genes produced no overt phenotype. We extended our sequence comparisons to include putative FRKs encoded in other plant sequenced genomes. We observed that different subgroups expanded subsequent to speciation.

**Conclusions:**

*Arabidopsis thaliana* as well as all other plant species analyzed contain multiple copies of genes encoding FRK activity. Sequence comparisons among multiple species identified a minimal set of three distinct FRKs present on all species investigated including a plastid-localized form. The selective expansion of specific isozymes results in differences in FRK gene number among species. AtFRKs exhibit substrate inhibition, typical of their mammalian counterparts with the single AtFRK1 lacking this property, suggesting it may have a distinct in vivo role. Results presented here provide a starting point for the engineering of specific FRKs to affect biomass production.

## Background

Fructose is an abundant sugar found in plants, generated via the breakdown of sucrose either by invertases, which hydrolytically produce fructose and glucose, or by sucrose synthases, reversibly producing fructose and NDP-glucose using a nucleoside diphosphate. To enter metabolism, fructose must first be phosphorylated most typically to fructose-6-phosphate (F6P) by a fructokinase (FRK, EC 2.7.1.4). Curiously, FRKs are members of a family of diverse kinases known as the phosphofructokinase B (pfkB) family based on their sequence similarity to the founding member, *E. coli* phosphofructokinase 2 (Pfk-2) which is the minor fructokinase isoform in this species [1]. The pfkB family of enzymes across kingdoms includes ribokinases, phosphofructokinases, adenosine kinases (ADKs) and several others with diverse substrate specificities [2].

Typical PfkB protein structure is a large domain consisting of a β-sheet sandwiched between several α-helices, and a smaller domain, known as the lid domain, comprised of another β-sheet attached to the larger domain by short loops that act as a hinge [3, 4]. The active site lies in a cleft between the two domains. Substrate binding induces a conformational change in which the lid closes over the substrates [4]. Catalysis occurs while the protein is in the closed state. After catalysis, the protein returns to an open state and the products are released [4]. Binding of substrates to pfkB proteins follows ordered bi-bi kinetics where the carbohydrate enters first, followed by ATP [5].

PfkB proteins possess two signature motifs: a di-gly (GG) motif in the N-terminal region and a G/AXGD motif in the C-terminal region [6]. The role of each motif has been identified through mutational and structural analyses. The GG motif provides flexibility in the hinge region that connects the lid and the large domain. Substitution of the second glycine to aspartate in the GG motif in the *Leishmania donovani* pfkB member ADK, decreased enzyme activity to less than 1% of that of the wild-type enzyme and reduced substrate binding affinity, presumably due to the inability of the mutant protein to adopt the closed conformation that supports catalysis [7]. The aspartate in the G/AXGD motif acts as a base during catalysis and activates the C6 fructose hydroxyl group for nucleophilic attack on the γ-phosphate in ATP [2, 4]. Mutation of the codon for aspartate to asparagine in *E. coli* Pfk-2 significantly abrogated enzymatic activity, but substrate binding was only mildly affected [2], demonstrating a key catalytic role for aspartate in the G/AXGD motif.

Many pfkB proteins are active as dimers generated through interactions between the lid domains of two monomers [3, 4]. Interactions between β-sheets are mainly hydrophobic and, interestingly, both β-sheets contribute a strand to the other β-sheet in what has been called a β-clasp [4]. ATP has been shown to lead to substrate inhibition of pfkB family members. Excess ATP binds an allosteric site and leads to the formation of tetramers, which are inactive, though it has been shown that substrate inhibition by ATP can still occur in proteins containing substitutions that prohibit formation of tetramers so the regulatory role of ATP is not completely understood [8–10]. Aside from their regulation by ATP, pfkB family enzymes are activated by both monovalent and divalent cationic cofactors. Potassium is thought to be the physiological monovalent cofactor. The binding of a potassium ion in the active site activates pfkB enzymes via a conformational shift that results in an anion hole [11]. The ATP-magnesium chelate is considered the actual substrate of pfkB enzymes and magnesium is postulated to aid in catalysis [12, 13].

Despite renewed interest in understanding regulation of carbon flux within the plant and overall plant biomass accumulation for energy purposes, biochemical studies on FRK activities have been characterized only in a few species, mostly tomato, potato and *Arabidopsis thaliana*. Among these species, FRK activities are best characterized in tomato and four tomato FRK isozymes have been studied functionally and biochemically [14]. As GFP fusions, tomato FRK3 localizes to plastid stroma and the other three FRKs localize to the cytosol [15]. Tomato plants with antisense RNAs targeting FRK1 or FRK2 had markedly less FRK activity than control plants [16]. Surprisingly, fructose levels were lower in all FRK knock-down (KD) plant lines compared to control plants. While the growth of all FRK KD plant lines analyzed in the aforementioned study were stunted, KD of FRK2 affected plant size more than FRK1. Subsequently FRK2 was shown to be involved with both xylem and phloem development [17]. Vascular cells were smaller in FRK2 KD plants than control plants and vessels in the stems were thinner, leading to impaired water conductance. RNAi-mediated KD of tomato FRK3 correlated with diminished stem xylem and reduced water conductance. Simultaneous KD of both FRK2–3 led to severe defects in plant growth that were more drastic than seen in FRK2 KD plants alone [18]. In experiments using the tomato *FRK4* promoter to drive expression of diphtheria toxin A in Arabidopsis to assess the tissue-specific expression profile of *FRK4*, David-Schwartz et al. found that the lethal effects of the toxin were limited to pollen and developing anthers suggesting *FRK4* expression is restricted to these organs [19]. The differences in tissue-specific expression and the differential phenotypes seen upon KD of different FRKs suggests non-overlapping roles for different FRKs in tomato.

The growth and vasculature defects in the tomato KD plants suggest that FRKs could be involved in cell wall development, which is a logical possibility for carbohydrate metabolizing enzymes. Sucrose synthase generates fructose and UDP-glucose, the latter of which is a precursor of cellulose, a major constituent of plant cell walls, and fructose is a feedback inhibitor of sucrose synthase. Since FRKs phosphorylate fructose, and thereby reduce intracellular fructose pools, they are hypothesized to indirectly affect cellulose production through modulation of sucrose synthase activity and UDP-glucose production. This hypothesis implicates FRKs as relevant to crop engineering for lignocellulosic biofuel production [20, 21]. In aspen, RNAi targeting *FRK2* led to a reduction in the UDP-glucose pool, which was accompanied by reduced cell wall fiber thickness and a lower proportion of cellulose in cell walls [21]. These data together with results from experiments in tomato suggest that further studies of FRKs may lead to a better understanding of, and advances in, the generation of cellulosic biomass.

In Arabidopsis, two electrophoretically distinct FRK activities have been identified and characterized biochemically for substrate specificities and sensitivities to ions and metabolites, though the identities of protein or proteins in each band are unknown [22]. In control experiments while studying proteins related to FRKs, Arsova et al. demonstrated FRK activity for a plastidic Arabidopsis pfkB protein, At1g66430, and denoted it as FRK3 due to sequence similarity to tomato FRK3 [23]. To explore the diversity of FRKs within a single species, we report here the identification of the FRK family in the model plant *Arabidopsis thaliana*, biochemical characterization of seven active members, and the identification of one pseudogene. All seven members phosphorylated fructose specifically, and henceforth will be referred to as FRKs. Fluorescently tagged Arabidopsis FRK enzymes localized to the cytosol, with the exception of At1g66430/FRK3, which was plastid-localized. Sequence analysis using predicted FRKs from other sequenced genomes support a minimal set of three types of FRKs in plants. Analysis of other plant species also suggests that some of the isozymes may have expanded after speciation. Arabidopsis lines with T-DNA-mediated disruption of single FRKs did not noticeably affect plant growth. The data presented in this manuscript represents the first steps in defining the biological and biochemical roles for the individual FRK enzymes in Arabidopsis.

## Results

### The Arabidopsis genome encodes seven functional FRKs

**Fig. 1:**
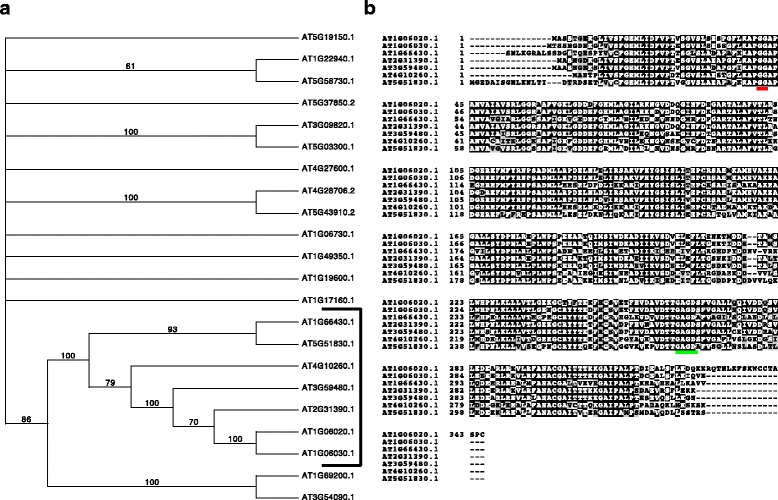
Identification of seven putative *Arabidopsis thaliana* (Col-0) FRK enzymes. **a** Consensus bootstrap phylogenetic tree of the 22 pfkB proteins in Arabidopsis. The seven Arabidopsis FRKs studied in this manuscript are bracketed. The tree shows the results of 100 bootstrap replicates of heuristic searches using maximum parsimony in PAUP. Bootstrap values are shown for clades with >50% support and clades with <50% were collapsed. **b** Alignment of seven putative Arabidopsis FRK protein sequences. *Black* and *grey boxes* indicate amino acids identical or with conservative substitutions in >50% of the proteins, respectively. The GG and G/AXGD motifs are *underlined* in *red* and *gree*n, respectively

Arsova et al. [23] determined that one member of the pfkB family of proteins in *Arabidopsis thaliana* ecotype Col-0, encoded in At1g66430, therein named FRK3, was an active FRK after expression of the recombinant protein in *E. coli*. To determine whether other pfkB enzymes in Arabidopsis are active FRKs, we first generated a phylogenetic tree based on an alignment of the amino acid sequences of the 22 putative pfkB family members in Arabidopsis to determine whether and which other pfkB proteins clade with FRK3 (Fig. 1a). Several clades with 1–2 members and one large sub-family were apparent. One clade contained the two characterized adenosine kinases, ADK1 (At3g09820) and ADK2 (At5g03300) [24]. Approximately half of the Arabidopsis pfkB proteins are un-characterized, so the functional significance of these phylogenetic groupings remains uncertain. The one large sub-family contained sequences from nine proteins divided into two branches. One branch within this clade comprised the two fructokinase-like proteins FLN1 (At3g59480) and FLN2 (At1g69200), for which no kinase activity has been detected [23]. The other branch comprised seven proteins including FRK3. An eighth putative pfkB family member was excluded from these analyses because it was found to be a pseudogene (discussed in the following paragraph). Two peptide sequences that were present in the FRK3 inclusive branch, At1g66430 and At4g10260, were both designated FRK3 in different publications [23, 25]. We have chosen to denote At1g66430 as FRK3 because of its similarity to the tomato FRK3 (Table 1) as previously noted [18]. At4g10260 is designated FRK4 here [25]. FRK1 (At5g51830) and FRK2 (At2g31390) were previously annotated due to their similarity to the tomato isozymes [25]. To visualize specific regions of highest identity we aligned the peptide sequences of the seven putative Arabidopsis FRK proteins (Fig. 1b). FRK3 was predicted to contain a chloroplast transit peptide (cTP) by the chloroP software [26] and the predicted cTP was removed for the alignment. 47% of the amino acids are identical between all seven members of the putative FRK branch. Both the GG and G/AXGD motifs, underlined in red and green respectively (Fig. 1b), were conserved in all members, and, notably, the G/AXGD motif was in the form GAGD in all members.

**Table 1:** Arabidopsis FRK nomenclature

AGI number	Previous designation (citation)	Designation in this manuscript
At5g51830	FRK1 (Pego and Smeekens)	FRK1
At2g31390	FRK2 (Pego and Smeekens)	FRK2
At1g66430	FRK3 (Arsova et al.)	FRK3
At4g10260	FRK3 (Pego and Smeekens)	FRK4
At1g06020	na	FRK5
At1g06030	na	FRK6
At3g59480	na	FRK7

**Fig. 2:**
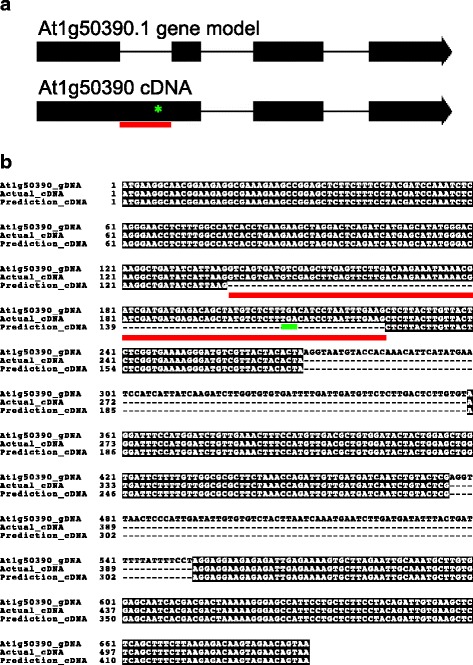
At1g59480 produces an mRNA encoding a non-functional protein. **a** Schematic of the At1g59480 predicted gene model (*top*) and experimentally determined cDNA (*bottom*). Exons and introns are represented as thick and thin lines, respectively. Intron 1 sequences in experimental cDNA are *underlined* in *red* and location of the predicted premature stop codon is denoted by a *green asterisk*. **b** Sequence alignment of At1g59480 genomic DNA (At1g59480_gDNA), experimentally determined cDNA (actual_cDNA), and predicted cDNA (prediction_cDNA) showing presence of DNA in the experimentally derived sequence corresponding to intron 1 *underlined* in *red*, in-frame stop codon *underlined* in *green*

Another predicted pfkB protein, At1g50390, claded with the FRK branch, but the predicted protein is only 16 kDa, while other FRKs range from ~34–41 kDa. The 16 kDa protein is missing at least half of the lid domain and alpha helices in the large domain. In addition, there is no evidence that this gene is expressed based on data from TAIR and Genvestigator [27]. According to the TAIR11 gene model, At1g50390 includes three introns (Fig. 2a), but two independent cDNAs synthesized from total Arabidopsis ecotype Col-0 seedling RNA contained sequences identical to the predicted first intron (Fig. 2a-b). As a result, the cDNA contains an in-frame stop codon encoded in the unspliced first intron. If this mRNA were translated, the protein would be 7.85 kDa and lack a functional lid and the catalytic residues. Based on these data we conclude that At1g50390 does not express a functional protein in Col-0 and, thus, have not included it in our phylogenetic or biochemical analyses.

cDNAs for the remaining seven predicted proteins were either isolated from Col-0 cDNA or obtained from the Arabidopsis Biological Resource Center and sequenced to verify the predicted ORFs. All seven ORFs matched the TAIR11 representative gene models, and contained both signature motifs characteristic of pfkB proteins.

### All seven putative FRKs are active and phosphorylate fructose specifically

**Fig. 3:**
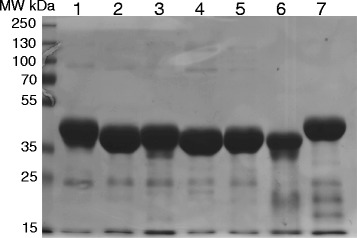
Purification of putative Arabidopsis FRK enzymes from *E. coli*. Coomassie brilliant *blue* stained gel of purified FRKs fractionated by 10% SDS-PAGE. Lanes were loaded with ~5 μg purified protein as follows: 1. At1g06020/FRK5; 2. At1g06030/FRK6; 3. mAt1g66430/FRK3; 4. At2g31390/FRK2; 5. At3g54090/FRK7; 6. At4g10260/FRK4; 7. At5g51830/FRK1. MW markers in kDa are shown

**Fig. 4:**
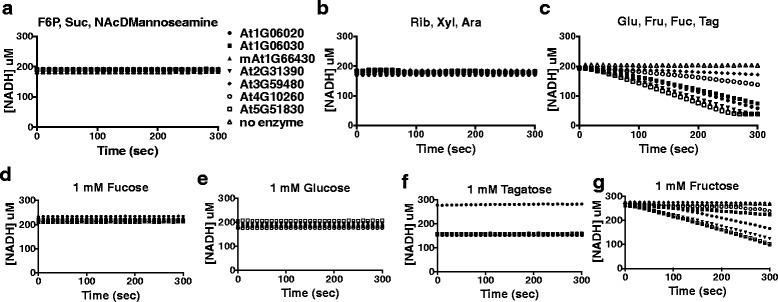
Putative Arabidopsis FRKs specifically phosphorylate fructose. Graphs of [NADH] vs time in coupled enzyme assays in the presence of carbohydrate mixes of (**a**) Fructose-6-phosphate (F6P), Sucrose (Suc), and N-Acetyl-D-mannoseamine (N-AcDMannosamine). **b** Ribose (Rib), Xylose (Xyl), and Arabinose (Ara). **c** Glucose (Glu), Fructose (Fru), Fucose (Fuc), and Tagatose (Tag). Graphs of [NADH] vs time in coupled enzyme assays in the presence of single carbohydrates (**d**) Fucose (**e**) Glucose (**f**) Tagatose and (**g**) Fructose. Experiments were repeated at least twice with two technical replicas per experiment

To verify whether the seven members of the putative FRK clade exhibit FRK activity, we expressed each protein in *E. coli* with 6xHis and FLAG epitope tags and purified them using Ni-agarose resin (Fig. 3). Since the substrate specificity of known pfkB family proteins varies greatly [2], we measured NADH oxidation as a proxy for FRK activity in the presence of an ATP regeneration system (see Experimental Procedures) in pools of carbohydrate substrates. No change in NADH concentration was seen for any of the proteins in a reaction mixture containing fructose-6-phosphate, sucrose, and N-acetyl-mannosamine suggesting that none of the putative FRKs could phosphorylate any of these carbohydrates (Fig. 4a). Similarly, the proteins were not active in the presence of ribose, xylose, and arabinose (Fig. 4b). In contrast, NADH oxidation was observed in the presence of glucose, fructose, fucose, and tagatose (Fig. 4c), suggesting that one or more of these sugars is a substrate. When these carbohydrates were parsed, fucose, glucose or tagatose alone produced no change in NADH concentration (Fig. 4d-f). However, all seven enzymes were active in the presence of fructose (Fig. 4g), suggesting that each of the seven enzymes possesses FRK activity and that their activity is specific for fructose. The two proteins that comprise the most closely related phylogenetic branch, FLN1 and FLN2, lack detectable fructokinase activity [23], and the only biochemically characterized enzyme in the unresolved polytomy of proteins related to the FRK clade, At1g17160, specifically phosphorylates ribose [28], suggesting that the seven FRKs identified herein likely comprise the Arabidopsis FRK family. As such, we propose the nomenclature in Table 1 based on previously defined nomenclature [23, 25] and degree of similarities among the Arabidopsis proteins, and we will use it in the remainder of this manuscript.

**Fig. 5:**
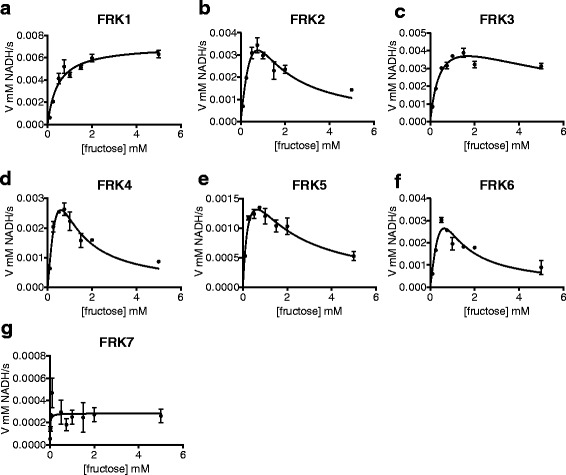
Most Arabidopsis FRKs exhibit substrate inhibition in high concentrations of fructose. Velocity vs substrate concentration plots for (**a**) FRK1 (**b**) FRK2 (**c**) FRK3 (**d**) FRK4 (**e**) FRK5 (**f**) FRK6 (**g**) FRK7. Curves were fit to either the Michaelis-Menten equation in the case of FRK1 or substrate inhibition for FRK2–7 in Prism. Kinetic assays were run in triplicate. Points represent the mean of three technical replicates and error bars indicate SEM

The same NADH oxidation assay was used to determine the kinetic parameters of each of the FRK enzymes for their substrates fructose and ATP (summarized in Table 2). The Km values were similar for FRK1–6 as they were all between 470 ± 77 μM and 230 ± 56 μM (Fig. 5a-g, Table 2), whereas FRK7, the least active enzyme exhibited the strongest binding affinity (Km = 12 ± 8 μM, Fig. 5g). Substrate inhibition was observed for all FRKs at levels of fructose above 1 mM with the exception of FRK1 and FRK7 (Fig. 5). The activity of FRK7 was much lower than the other FRKs and, as such, the activity measurements produced more experimental variation, which may have masked its inhibition by fructose.

**Table 2:** Biochemical parameters determined for Arabidopsis FRK isozymes

Enzyme	Km μM Fructose	Km μM ATP	Fruc substrate inh.	Vmax (μM product *min^−1^ *mg enzyme^−1^)	kcat (s^−1^)	Catalytic efficiency kcat/Km (ATP)
FRK1	470 +/− 77	52 +/− 7.8	−	2.3E + 05	14.2	2.7E + 05
FRK2	370 +/− 91	85 +/− 8.7	+	1.8E + 05	10.3	1.2E + 05
FRK3	480 +/− 99	52 +/− 9.8	+	2.1E + 05	14.3	2.7E + 05
FRK4	230 +/− 56	95 +/− 7.9	+	1.9E + 05	11.1	1.2E + 05
FRK5	320 +/− 89	180 +/− 57	+	7.7E + 04	4.8	2.7E + 04
FRK6	260 +/− 81	160 +/− 21	+	2.1E + 05	12.8	8.0E + 04
FRK7	12 +/− 8.4	280 +/− 94	−	2.6E + 04	1.5	5.4E + 03

**Fig. 6:**
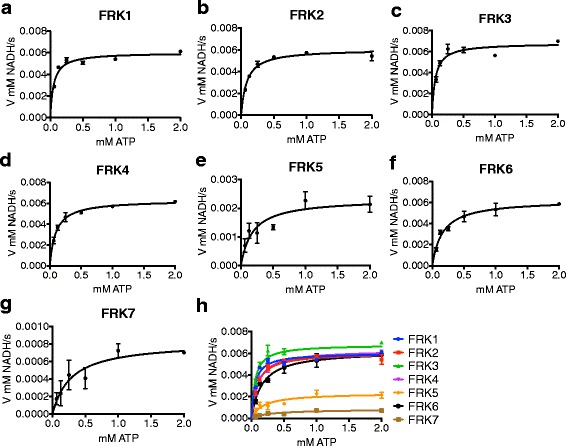
Arabidopsis FRKs exhibit Michaelis-Menten kinetics in varied ATP. Velocity vs substrate concentration plots for (**a**) FRK1 (**b**) FRK2 (**c**) FRK3 (**d**) FRK4 (**e**) FRK5 (**f**) FRK6 (**g**) FRK7. Data were plotted on the same graph in *H*. for direct comparison. *Curves* were fit to Michaelis-Menten equation via non-linear curve fitting in Prism. Kinetic assays were run in triplicate. *Points* represent the mean of three technical replicates and error bars indicate SEM

As demonstrated for other plant FRKs [22, 29], most of the Arabidopsis FRK enzymes were inhibited by higher concentrations of fructose (Fig. 5a-h, Table 2). Km_ATP_ values were also very similar and inhibition by ATP was not apparent for any of the FRK enzymes (Fig. 6a-h, Table 2). As such we calculated Vmax and k_cat_ values under varied concentrations of ATP under a constant concentration of fructose (Table 2). Calculated Km and k_cat_ values were also used to calculate the catalytic efficiency of the FRKs (Table 2). Two groups with different amounts of activity were apparent (Fig. 6h). The group with higher activity included FRK1–4 and FRK6; while the group with lower activity included FRK5 and FRK7 (Fig. 6h). The group with higher activity had similar k_cat_ values, which varied between 10.3 and 14.3 s^−1^, whereas the group with lower activity had k_cat_ values of 4.8 s^−1^ and 1.5 s^−1^ for FRK5 and FRK7, respectively (Table 2).

**Fig. 7:**
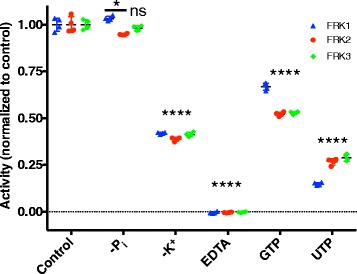
A subset of Arabidopsis FRKs shares biochemical cofactor requirements. Scatterplots comparing enzyme activity of FRK1 (*blue*), FRK2 (*red*), and FRK3 (*green*) in various conditions normalized to that of their respective control experiment. Control experiments were carried out in a complete mixture as compared to mixtures where inorganic phosphate was omitted (−Pi), K+ was replaced with Na + to preserve ionic strength (−K+), Mg2+ was omitted and replaced with EDTA (EDTA), or ATP was replaced with either GTP (GTP) or (UTP). Statistical analysis was conducted with Dunnett’s post hoc test to compare the mean of each enzyme in each condition to the mean of the control experiment for the corresponding enzyme. *p < 0.05, ****p < 0.0001, ns = not significant. Experiments were carried out in quadruplicate, all data points are shown

We then assessed the cofactor requirements of a subset of Arabidopsis FRKs, FRK1–3. Unlike other pfkB proteins, most notably the ribokinases and adenosine kinases [30, 31], Arabidopsis FRK1–3 were not significantly sensitive to the absence of inorganic phosphate from the reaction mixture (Fig. 7). Because potassium ions activate pfkB family members via creation of an anion hole in their active site [11], we tested whether Arabidopsis FRK1–3 were activated to the same extent upon the substitution of sodium for potassium as the monovalent cation in the reaction mixture. Replacement with sodium resulted in the reduced activity of FRK1–3 by roughly 60% of their respective activities in the presence of potassium (Fig. 7). Since magnesium is known to be required for ATP-dependent phosphorylation reactions we next, tested whether or not magnesium is required for FRK activity. Magnesium was omitted from the reaction mixture and EDTA was added to chelate any magnesium that may have co-purified with the enzymes. FRK1–3 activity was completely lost in the reaction mixture containing EDTA (Fig. 7). Other FRK enzymes can use other nucleotide triphosphates besides ATP as kinase substrates, albeit with decreased effectiveness [22]. Accordingly, FRK1 activity decreased by 35% activity in the presence of 1 mM GTP (Fig. 7), while FRK2–3 activities were approximately halved (Fig. 7). Substitution of ATP with 1 mM UTP resulted in the loss of 85% and approximately 75% of FRK1 and FRK2–3 activities, respectively (Fig. 7).

### Single T-DNA insertional mutants exhibit no overt growth phenotype

**Fig. 8:**
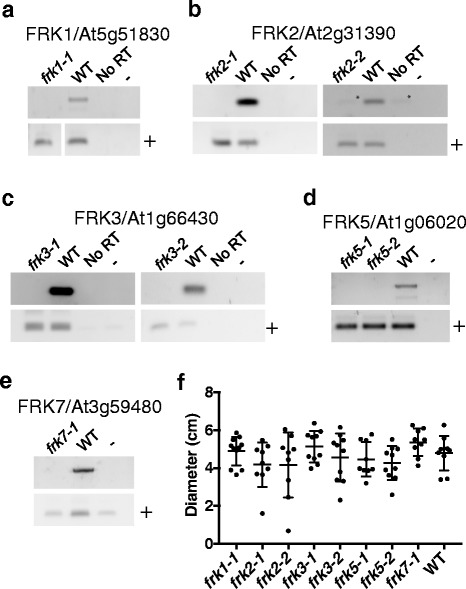
Single T-DNA insertional mutants exhibit no growth phenotype. RT-PCR results on cDNAs generated from seedling mRNA using primers flanking the T-DNA insertional site for (**a**) *frk1–1,* (**b**) *frk2–1* and *frk2–2,* (**c**) *frk3–1* and *frk3–2,* (**d**) *frk5–1* and *frk5–2*, and (**e**) *frk7–1* and Col-0 (WT). Primers specific to another unrelated pfkB family member were also used in all cases to verify presence of cDNA in all reactions, denoted as +. *F*. Scatterplot of plant diameters at 21 days of growth. Measurements were made in ImageJ and plotted in Prism. Growth experiments were repeated twice with the exception of *frk5–2*, which was repeated once. All data points are shown from one representative experiment, the independent experiment gave the same result. ANOVA analysis was carried out in Prism. There were no statistical differences between any of the rosette diameters

To further our understanding of the in vivo role of FRK enzymes in Arabidopsis, we acquired Arabidopsis plants harboring T-DNA-mediated gene disruption mutants for five of the seven FRKs (T-DNA insertional mutants for FRK4 and FRK6 were unavailable). Each mutant line was confirmed homozygous for the T-DNA insertion in the respective gene (data not shown) and none of the T-DNA insertional mutants made authentic transcripts for their respective disrupted FRK gene (Fig. 8a-e). After 21 days of growth on soil there was no difference in rosette diameter among any of the single FRK mutants or WT control plants (Fig. 8f), suggesting a lack of vegetative phenotype and possible redundancy between some of the FRKs.

### Subcellular localization of Arabidopsis FRKs

**Fig. 9:**
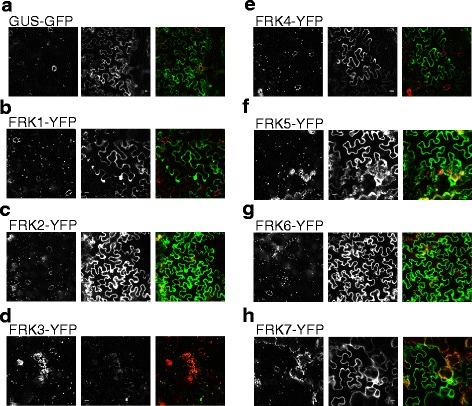
Localization of fluorescently tagged Arabidopsis FRKs transiently expressed in tobacco leaves. Representative images of chlorophyll auto-fluorescence (*left panel*), YFP or GFP-fluorescence (*center panel*), and chlorophyll (*red*) and YFP- or GFP- fluorescence (*green*) overlaid (*right panel*) of (**a**) GUS-GFP (**b**) FRK1-YFP (**c**) FRK2-YFP (**d**) FRK3-YFP (**e**) FRK4-YFP (**f**) FRK5-YFP (**g**) FRK6-GFP (**h**) FRK7-YFP. Scale = 10 μM

To gain further insight into the biological roles for Arabidopsis FRK enzymes, we visualized the intracellular localization of FRKs expressed as C-terminal yellow fluorescent protein (YFP) fusions transiently in tobacco leaves (*N. benthamiana*) and examined by confocal microscopy. The majority of a plant cell’s cytoplasm comprises the vacuole, such that the cytosol is compressed between the vacuolar and plasma membranes. Cytosolic proteins therefore appear around the edges of cells by microscopy, as seen for a known GFP-tagged cytosolic protein, β-glucuronidase (Fig. 9a). FRK1-YFP was observed in the cytosol as the YFP signal was most abundant around the edges of cells with apparent cytosolic bridges connecting parts of the cell (Fig. 9b). The same was true for FRK2-YFP, and FRK4–7-YFP (Fig. 9c, e-h). As mentioned previously, FRK3 was predicted to contain a cTP and, therefore, predicted to be chloroplast-localized. We found FRK3-YFP fluorescence to be coincident with chlorophyll auto-fluorescence, which confirms its predicted chloroplast localization (Fig. 9d).

### Other plant species also encode multiple FRKs

In order to determine whether orthologous FRKs are present in other plant species for all seven Arabidopsis FRKs, we compared the amino acid sequences of the seven active Arabidopsis FRKs with those of other plant species with sequenced genomes including *Medicago truncatula*, *Oryza sativa* (rice), *Solanum lycospersicum* (tomato), *Populus trichocarpa* (poplar), *Zea mays* (maize), *Brachypodium distaychon*, and *Physcomitrella patens* (moss). We then created a phylogenetic tree using all protein sequences along with all Arabidopsis pfkB sequences. We included all proteins that were within one branch from any of the seven active Arabidopsis FRKs. This yielded 51 protein sequences comprised of seven from Arabidopsis, 11 from Medicago, three from rice, four from tomato, four from Populus, four from Brachypodium, eight from maize, and eight from Physcomitrella (moss).

**Fig. 10:**
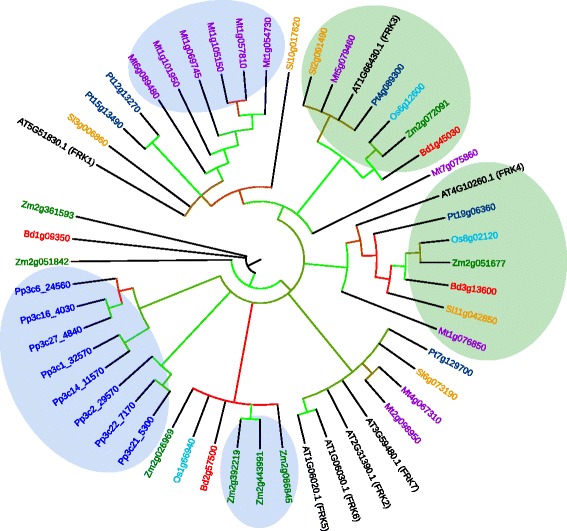
Phylogeny of putative FRK enzymes from multiple species. Consensus bootstrap phylogenetic tree of putative FRK proteins from *Medicago truncatula* (Mt, *purple*), *Oryza sativa* (Os, *teal*), *Solanum lycopersicum* (Sl, *orange*), *Populus trichocarpa* (Pt, *dark blue*), *Physcomitrella patens* (Pp, *blue*), *Zea mays* (Zm, *green*), and *Brachypodium distachyon* (Bd, *red*) related to Arabidopsis FRKs (*black*). Clades containing putative paralogs are highlighted in *blue*. Clades containing at least one putative ortholog from all species except Physcomitrella are highlighted in *green*. The tree shows the results of 100 bootstrap replicates. Bootstrap values are depicted in clades with >50% support by colored edges, with *green* and *red* representing bootstrap support close to 100% and 50%, respectively, and intermediate colors representing values between 50 and 100%. Clades with <50% support were collapsed

Several interesting relationships were apparent from the resulting phylogenetic tree comprised of the 51 confirmed and putative FRKs (Fig. 10). All Physcomitrella sequences were confined to two clades and, interestingly, those clades included Physcomitrella sequences only, indicating that Physcomitrella FRK sequences are more similar to each other than they are to FRKs from other species. There were two clades that included a single sequence from all other species excluding Physcomitrella (Fig. 10, green shading). One clade contains AtFRK3 and the second includes AtFRK4. Another large clade includes four Arabidopsis proteins, FRK2, FRK5–7, as well as sequences from poplar, tomato, and Medicago, but does not include any sequences from a monocot species. Similarly, another clade contains FRK1, and a tomato sequence, as well as two poplar sequences, and six Medicago sequences that are more distantly related, and no monocot proteins. There is one weakly separated monocot-specific clade with four maize sequences and one each from rice and Brachypodium. A few monocot proteins seem equidistant from all other pfkBs.

## Discussion

Fructose is one of the major sugars found in plant cells as it is a common product of the two major sucrose cleaving enzymes, and it must be phosphorylated to enter metabolism as F6P. Here we provide evidence that there are at least seven active fructokinase isozymes in the model plant Arabidopsis. This trend of multiple FRKs also appears to extend to other plant species as there were at least three putative FRKs encoded by the genome of each species included in our analysis. The Physcomitrella genome encodes eight putative FRKs, which clade together but group away from all other species in our analysis. The fewest FRKs were identified in rice and we speculate that the three putative rice FRK enzymes may represent the minimum FRK cohort that supports plant survival. The groups of FRK paralogs in Arabidopsis, Physcomitrella, and Medicago that are more closely related to each other than those of any of the other species in our analysis suggest that each has a common ancestral gene that then may have duplicated multiple times after speciation.

The similarity between the Arabidopsis FRK protein sequences suggested that their biochemical activity would likely be very similar. Indeed, each of the Arabidopsis FRKs in our analysis phosphorylated fructose specifically. Five Arabidopsis FRKs were roughly equivalent in activity while the other two were significantly lower. The lower activities of FRK5 and FRK7 is surprising given the sequence similarities between these two and the other FRKs. Upon closer inspection of the peptide sequences there are no amino acid or motif differences that are shared between only FRK5 and FRK7, leaving the molecular explanation for the difference in activity as compared to the other FRKs unresolved. The regulation of most Arabidopsis FRKs, with the exception of FRK1 that lacks apparent substrate inhibition by fructose, also appears to be very similar, though the mechanism of substrate inhibition in FRK enzymes by fructose remains unknown. The *E. coli* PFK-2 shows substrate inhibition by ATP, which involves an allosteric ATP binding site made up partly by the first ATP molecule bound to the protein [9]. The structures of several pfkB family members have been determined and, consistent with the known ordered bi-bi reaction mechanism, the binding site for the carbohydrate ligand is deep in the cleft between the lid and αβα domains and would be occluded if ATP, which binds closer to the surface, bound first [4]. Allosteric substrate inhibition by ATP is a property of other pfkB enzymes such as ribokinases and PFK-2 [8–10], though residues that interact with the allosteric ATP in PFK-2 [10] are not conserved in any of the Arabidopsis FRKs studied herein. This finding explains why the Arabidopsis FRKs do not appear to be inhibited by ATP. However, it is possible that an allosteric fructose binds in an analogous way to the allosteric ATP in PFK-2, though currently there is no structural or biochemical data to support this hypothesis.

The ionic cofactor requirements for the tested Arabidopsis FRKs are comparable to those of characterized pfkB family enzymes from other species, which further supports that potassium and magnesium are general cofactors for pfkB family enzymes [11–13]. Inorganic phosphate activates ribokinase (RBSK) enzymes [13, 30, 31] and was included in our reactions to determine the kinetic parameters of the Arabidopsis FRKs. However, inorganic phosphate was not required for FRK activity, which suggests that activation by inorganic phosphate may be specific to RBSK enzymes rather than a general characteristic of the pfkB family. FRK activity was diminished but detectable upon the replacement of ATP with either GTP or UTP. This demonstrates that, like previously characterized FRK enzymes, Arabidopsis FRKs are able to utilize other nucleoside triphosphates as phosphate donors [22], though ATP is likely their preferred substrate.

An important point to consider as it pertains to the possible redundancy between the Arabidopsis FRKs is their tissue-specific expression profiles. In their manuscript describing the tomato *FRK4*, David-Schwartz et al. [19] discussed publicly available Arabidopsis microarray data for four of the FRKs characterized in this manuscript. Briefly, *FRK1* and *FRK3* were expressed at moderate to high levels in all plant tissues with the exception of stamens where their expression was low. *FRK7* was expressed at high levels in pollen and in roots, and was also present in siliques and stems. For *FRK4*, which they report to be the likely functional Arabidopsis homolog of tomato *FRK4*, expression was confined to mature pollen. Examining the tissue specific expression profiles from the database used in the aforementioned study, Arabidopsis *FRK5* and *FRK6* are both expressed only at low levels. *FRK5* is expressed mainly in roots, flowers, and seeds, while *FRK6* is expressed at similar low levels in roots, flowers, and pollen. *FRK2*, on the other hand, was expressed at moderate levels throughout the plant and expressed highly in hypocotyls, roots, and stems. The ubiquitous and generally moderate to high levels of expression for FRK1, FRK2, and FRK3 further suggests that these enzymes may exhibit functional redundancy. The low level of expression of *FRK5* and *FRK6* in any tissue suggests that they play minor roles in plant metabolism. We did not examine the effects of the loss of *FRK4* as no T-DNA lines were available when we began these studies. However, we speculate that the loss of FRK4 may result in pollen deficiencies due to the tissue specific expression of its putative tomato ortholog [19].

The biochemical data presented here combined with the similar subcellular localization for six of the seven Arabidopsis FRKs further suggest possible redundancies between two or more Arabidopsis FRKs. Indeed, we observed no discernable differences in the vegetative growth upon T-DNA-mediated inactivation of Arabidopsis *FRK1*, *FRK2*, *FRK5* or *FRK7*. FRK3 is the only isozyme that is differentially localized, so we expected to see a phenotypic difference upon its inactivation. However, growth of *FRK3* T-DNA line was also not different than wild type.

The role of plastidic FRK is unknown. In prokaryotes it is well established that fructose in the unphosphorylated form will more easily pass through cell membranes. So, unphosphorylated plastidic fructose may also freely cross the chloroplast membrane making it available for phosphorylation by a cytosolic FRK. This scenario explains why the *FRK3* mutant may not exhibit a growth phenotype but also does not explain the possible importance of plastidic FRK.

Currently, there is no evidence that FRKs, themselves, are signal transducers (reviewed in [32, 33]). However, there is evidence of fructose-mediated signaling, which could implicate FRKs in plant carbohydrate signaling. The addition of psicose, a C3 epimer of fructose that can be phosphorylated by FRKs, to growth media inhibited root growth in both lettuce and Arabidopsis [34, 35]. The inhibition of root growth by psicose was similar to inhibition by mannose through the known hexokinase (HXK) pathway [35]. Interestingly, psicose-mediated inhibition of root growth was shown to act independently of the HXK pathway [35]. Furthermore, the addition of fructose resulted in the loss of psicose-mediated inhibition [34]. Fructose, itself, acts as a signaling molecule through FRUCTOSE INSENSITIVE 1 (FINS1) and the transcription factor FSQ6/ANAC089, both of which, in turn, are linked to abscisic acid signaling in plants [36, 37]. Whether acting directly through an as yet unknown mechanism or indirectly via their effect on intracellular fructose pools, it is clear that in addition to the assimilation of fructose produced from sucrose, FRKs have some role in plant signaling.

Knockdown experiments in tomato and aspen suggest that FRKs could participate in regulating cell wall development, likely by modulating the flux of cellulose precursors. This implicates FRKs as possible targets for biofuel crop engineering. Mukherjee et al. (19) sought to increase cotton fiber yield via the overexpression of tomato FRK1. They noted an increase in seed cotton production, though much of the increase was likely due to an increase in seed number. The transgenic cotton had reduced sucrose levels, and, curiously, increased stem diameters, though they speculate that other factors could have affected stem diameter [20]. Although an increase in seed number did not fulfill the initial goal, this result suggests that characterizing FRKs in dual-use food and biomass crops such as sorghum could be of particular interest. Our analysis could provide a starting point for engineering of specific FRKs for utilization in such experiments.

## Conclusions

This manuscript describes the identification and initial biochemical characterization of seven active fructokinases (FRKs) in the model plant *Arabidopsis thaliana*. FRKs represent an important group of enzymes that maintain the metabolic flux of fructose, one of the breakdown products of sucrose, the mobile sugar in plants. A large subfamily of pfkB family genes with high peptide sequence similarity was identified that included eight putative enzymes. One was found to likely encode a pseudogene, as its cDNA product contained an unspliced intron that contained a premature stop codon. The remaining seven were bacterially expressed and found to phosphorylate fructose specifically with roughly similar kinetic parameters. All were cytosolic except one, which was plastidic. Inactivation of any of five of the seven for which T-DNA insertion mutants were available resulted in no overt phenotype. Sequence analysis, when extended to other species, suggests that the pattern of encoding several FRKs is a general property of plants. Our study represents the first side-by-side characterization of the FRK family as a whole in a plant species and provides the basis for further studies linking FRK activity through sucrose metabolism to the agronomically important area of cellulosic biomass production.

## Methods

### Chemicals

All chemicals were obtained from Sigma-Aldrich unless otherwise noted.

### Plant material and growth conditions

*Arabidopsis thaliana* Col-0 ecotype seeds (originally from the Arabidopsis Biological Resource Center (ABRC) and propagated in the laboratory) were surface sterilized in a solution of 30% bleach and 0.1% Triton X-100 for 10 min, rinsed with water, stratified for 48 h at 4 °C and grown for 10 days on solid (0.8% bacto-agar, Difco) germination media (GM) (4.3 g/l Murashige and Skoog (MS) basal salts, 2.5 mm MES, 1× B vitamins (0.5 μg/ml nicotinic acid, 1.0 μg/ml thiamine·HCl, 0.5 μg/ml pyroxidine·Cl, 0.1 μg/ml myo-inositol), 1% *w*/*v* sucrose (Fisher), pH 5.7). All plate-grown seedlings were grown at 20 °C under constant white light at 40–50 μ mol sec^−1^ m^−2^ and used for RNA isolation.

For phenotypic analysis of T-DNA insertional mutants, *FRK1*, SALK_046463 (*frk1–1*); two alleles for *FRK2*, SALKseq_17726 (*frk2–1*) and SALK_114786 (*frk2–2*); two alleles for *FRK3*, SALK_044085 (*frk3–1*) and SALK_035386 (*frk3–2*); two alleles for *FRK5*, SALK_027635 (*frk5–1*) and SALK_057002 (*frk5–2*); and one allele for *FRK7*, GABI_253H07 (*frk7–1*), seeds were obtained from ABRC and sown directly on soil, thinned to 1 plant per 8 cm by 8 cm square pot, with multiple pots for each genotype distributed among 4–7 trays and grown at 22 °C in cycles of 16 h light and 8 h dark (average 116 μmol⋅sec^−1^⋅m^−2^). Untransformed Col-0 was grown as control. The growth experiment was performed twice with identical results. Rosette size at 3 weeks was measured with Image J software from photographs, graphed and analyzed by one-way ANOVA with post-hoc tests in Prism (GraphPad). Genomic DNA isolation and PCR genotypic were performed as described previously [38]. Primers used are listed in Table 4.

*Nicotiana benthamiana seeds* were obtained from Bo Liu (Department of Plant Biology, from Valerie Williamson, Department of Plant Pathology and Nematology UC-Davis [39]) and grown in soil at 20 °C 18h day/night for 4 weeks. Fully expanded leaves were used.

### Protein sequence analysis

Full-length protein sequences of Arabidopsis FRKs were aligned using MUSCLE [40] and visualized using Boxshade. Gene identifiers for all proteins analyzed are listed in Table 3. Phylogenetic relationships between the 22 Arabidopsis pfkB-type proteins or sequences of putative FRKs from other species were evaluated by maximum parsimony in PAUP*4.0b10 [41] using an alignment of pfkB protein sequences constructed with MUSCLE [40] and manually adjusted to remove phylogenetically uninformative sequences. Statistical support for relationships was evaluated by 100 bootstrap replicates for the phylogram in Fig. 1, with ten random addition heuristic searches using the branch and bound algorithm for each bootstrap replicate using PAUP*4.0b10. For the generation of the phylogram in Fig. 10, Phytozome was used to search the genomes of *Medicago truncatula*, *Oryza sativa*, *Solanum lycopersicum*, *Populus trichocarpa*, *Physcomitrella patens*, *Zea mays*, and *Brachypodium distachyon* for the keyword “pfkB”. All resulting sequences were downloaded and aligned along with the peptide sequences from seven Arabidopsis FRKs in MUSCLE [40]. Then a phylogenetic tree was calculated based on that alignment in PhyML 3.0 [42] and visualized in iTOL [43]. Sequences that were within one branch of Arabidopsis FRKs were considered to be putative FRKs. All putative FRKs were realigned in MUSCLE [40] and the alignment was manually adjusted using jalview [44]. The phylogenetic tree in Fig. 10 was calculated from the trimmed alignment of putative and known FRKs from all species in PhyML 3.0 using default settings. Statistical support for relationships was evaluated by 100 bootstrap replicates in PhyML 3.0 and visualized in iTOL. Branches with >50% bootstrap support are depicted on the phylogram as colored edges where red represents those close to 50% and green represents those approaching 100%.

**Table 3:** Gene identifiers for those used in sequence analysis in Figs. 1 and 10

Species	Gene identifier (Phytozome)
*Arabidopsis thaliana*	At1g06020.1
*Arabidopsis thaliana*	At1g06030.1
*Arabidopsis thaliana*	At1g06730.1
*Arabidopsis thaliana*	At1g17160.1
*Arabidopsis thaliana*	At1g19600.1
*Arabidopsis thaliana*	At1g22940.1
*Arabidopsis thaliana*	At1g49350.1
*Arabidopsis thaliana*	At1g50390.1
*Arabidopsis thaliana*	At1g66430.1
*Arabidopsis thaliana*	At1g69200.1
*Arabidopsis thaliana*	At2g31390.1
*Arabidopsis thaliana*	At3g09820.1
*Arabidopsis thaliana*	At3g54090.1
*Arabidopsis thaliana*	At3g59480.1
*Arabidopsis thaliana*	At4g10260.1
*Arabidopsis thaliana*	At4g27600.1
*Arabidopsis thaliana*	At4g28706.1
*Arabidopsis thaliana*	At5g03300.1
*Arabidopsis thaliana*	At5g37850.1
*Arabidopsis thaliana*	At5g43910.1
*Arabidopsis thaliana*	At5g51830.1
*Arabidopsis thaliana*	At5g58730.1
*Oryza sativa*	LOC_Os08g02120.1
*Oryza sativa*	LOC_Os01g66940.1
*Oryza sativa*	LOC_Os06g12600.1
*Solanum lycopersicum*	Solyc11g042850.1.1
*Solanum lycopersicum*	Solyc06g073190.2.1
*Solanum lycopersicum*	Solyc03g006860.2.1
*Solanum lycopersicum*	Solyc10g017620.2.1
*Solanum lycopersicum*	Solyc02g091490.2.1
*Populus trichocarpa*	Potri.019 g063600.1
*Populus trichocarpa*	Potri.007 g129700.1
*Populus trichocarpa*	Potri.012 g132700.1
*Populus trichocarpa*	Potri.015 g134900.1
*Populus trichocarpa*	Potri.004 g089300.1
*Medicago truncatula*	Medtr1g076850.1
*Medicago truncatula*	Medtr2g098950.1
*Medicago truncatula*	Medtr4g067310.1
*Medicago truncatula*	Medtr1g054730.1
*Medicago truncatula*	Medtr1g057810.1
*Medicago truncatula*	Medtr1g105150.1
*Medicago truncatula*	Medtr1g069745.1
*Medicago truncatula*	Medtr1g101950.1
*Medicago truncatula*	Medtr6g089480.1
*Medicago truncatula*	Medtr7g075860.1
*Medicago truncatula*	Medtr5g079460.1
*Physcomitrella patens*	Pp3c22_7170V3.1.p
*Physcomitrella patens*	Pp3c21_5300V3.1.p
*Physcomitrella patens*	Pp3c2_29570V3.1.p
*Physcomitrella patens*	Pp3c16_4030V3.1.p
*Physcomitrella patens*	Pp3c27_4840V3.1.p
*Physcomitrella patens*	Pp3c6_24560V3.1.p
*Physcomitrella patens*	Pp3c1_32570V3.1.p
*Physcomitrella patens*	Pp3c14_11570V3.1.p
*Brachypodium distaychon*	Bradi1g45030.2.p
*Brachypodium distaychon*	Bradi3g13600.1.p
*Brachypodium distaychon*	Bradi1g09350.3.p
*Brachypodium distaychon*	Bradi2g57500.1.p
*Zea Mays*	GRMZM2G072091_P01
*Zea Mays*	GRMZM2G051677_P02
*Zea Mays*	GRMZM2G361593_P01
*Zea Mays*	GRMZM2G443991_P02
*Zea Mays*	GRMZM2G392219_P01
*Zea Mays*	GRMZM2G086845_P01
*Zea Mays*	GRMZM2G026969_P01
*Zea Mays*	GRMZM2G051842_P02

### Cloning

For *FRK2*, *FRK3*, *FRK4*, *FRK5*, and *FRK7*, RNA was extracted from 7-day-old Arabidopsis seedlings with the RNeasy Plant Mini Kit (Qiagen) and used to generate cDNA using the SuperScript III First-Strand Supermix (Invitrogen). pENTR223 and pDONR221 based clones for *FRK1* (G09822) and *FRK5* (DQ056446), respectively, were obtained from the ABRC and were recombined directly into destination vectors for bacterial expression. Clones for transient expression in tobacco leaves and for Arabidopsis transformation had their stop codon mutated and Gateway (Invitrogen) recombination sites added via PCR using the primers listed in Table 4. All PCR for cloning was carried out using Phusion high fidelity DNA polymerase (Fisher) according to the manufacturer’s instructions. PCR products for all clones were recombined into pDONR201 or pDONR207 (Invitrogen) via the BP clonase (Invitrogen) reaction and transformed into *E. coli* strain DH5α (New England Biolabs) for propagation of plasmid DNA. Sequences were verified at the UC Davis DNA sequencing facility. Clones were then recombined into pEAK2 [45] for bacterial expression; or pEARLEYGATE101 [46] or pGWB441 [47] via the LR clonase (Invitrogen) reaction for transient expression in tobacco leaves.

**Table 4:** Primers used in these studies

Cloning primers		
AGI Number of target and primer direction	Purpose	Primer sequence (5′- -3′)
At2g31390 – forward	FRK2 bacterial/plant expression	GGGGACAAGTTTGTACAAAAAAGCAGGCTTGATGGCATCCAACGGTGATAAAGG
At2g31390 – reverse	FRK2 bacterial expression	GGGGACCACTTTGTACAAGAAAGCTGGGTCCTATTTCTTTTCGAG
At1g66430 – forward	FRK3 bacterial expression/removal of cTP	GGGGACAAGTTTGTACAAAAAAGCAGGCTTGTCTAATCTCAAAGGAAGAGC
At1g66430 – reverse	FRK3 bacterial expression	GGGGACCACTTTGTACAAGAAAGCTGGGTCTTAAACGACGGCTTTGAG
At4g10260 – forward	FRK4 bacterial/plant expression	GGGGACAAGTTTGTACAAAAAAGCAGGCTTGATGGCTAATACTCCATTG
At4g10260 – reverse	FRK4 bacterial expression	GGGGACCACTTTGTACAAGAAAGCTGGGTCCTATTTAGACTTAGATTTC
At1g06030 – forward	FRK6 bacterial/plant expression	GGGGACAAGTTTGTACAAAAAAGCAGGCTTGATGACGTCATCCAACGGCG
At1g06030 – reverse	FRK6 bacterial expression	GGGGACCACTTTGTACAAGAAAGCTGGGTCCTATTCTACTTGTATCTTAAG
At3g59480 – forward	FRK7 bacterial/plant expression	GGGGACAAGTTTGTACAAAAAAGCAGGCTTGATGGCTGCATCTAACGGCGAG
At3g59480 – reverse	FRK7 bacterial expression	GGGGACCACTTTGTACAAGAAAGCTGGGTCTTAGTTTCCTTTCAGGAGGC
At5g51830 – forward	FRK1 plant expression	GGGGACAAGTTTGTACAAAAAAGCAGGCTTGATGGGTGAGGATGCAATCTC
At5g51830 – reverse	FRK1 plant expression stop codon mutation	GGGGACCACTTTGTACAAGAAAGCTGGGTCTGACGATCGAGTAGAAGAAAG
At2g31390 – reverse	FRK2 plant expression stop codon mutation	GGGGACCACTTTGTACAAGAAAGCTGGGTCCCATTTCTTTTCGAG
At1g66430 – forward	FRK3 plant expression	GGGGACAAGTTTGTACAAAAAAGCAGGCTTGATGGCTCTCCAAGCCACTAC
At1g66430 – reverse	FRK3 plant expression stop codon mutation	GGGGACCACTTTGTACAAGAAAGCTGGGTCTGAAACGACGGCTTTGAG
At4g10260 – reverse	FRK4 plant expression stop codon mutation	GGGGACCACTTTGTACAAGAAAGCTGGGTCCGATTTAGACTTAGATTTC
At1g06020 – forward	FRK5 plant expression	GGGGACAAGTTTGTACAAAAAAGCAGGCTTGATGGCATCATCCACCGGCG
At1g06020 – reverse	FRK5 plant expression stop codon mutation	GGGGACCACTTTGTACAAGAAAGCTGGGTCTGAACAAGGgctAGCCGTACAACACC
At1g06030 – reverse	FRK6 plant expression stop codon mutation	GGGGACCACTTTGTACAAGAAAGCTGGGTCCGATTCTACTTGTATCTTAAG
At3g59480 – reverse	FRK7 plant expression stop codon mutation	GGGGACCACTTTGTACAAGAAAGCTGGGTCTGAGTTTCCTTTCAGGAGGC
Primers for analysis of T-DNA insertional mutants		
Arabidopsis T-DNA line	Primer target	Primer sequence (5′- -3′)
*frk1–1* (SALK_046463)	gDNA	CTGGGATGAGATTCGACCAT
	gDNA/T-DNA	GGCAAGGTTCCTCAATCAAA
	cDNA flanking insert	ATGTTAGCTGATATTCTAAG
	cDNA flanking insert	TAGCAGCTTCTTCTGATGGC
*frk2–1* (SALKseq_17726)	gDNA/T-DNA	TCATGGCGAGGATCTTTTGCT
	gDNA	CACACTAGTTTTGCCTTCTGGT
	cDNA flanking insert	TAGTGGAGCCGTGTAGGTCA
	cDNA flanking insert	AGCTCAACATCGCTCACCTT
*frk2–2* (SALK_114786)	gDNA 5’	TTCTTCTCCTTGTCCTTCCTT
	gDNA/ T-DNA 3’	CGAGACGAGAAACAGCGATT
	cDNA flanking insert	CTTCGGCGAGATGCTAATCG
	cDNA flanking insert	TCCCAGCCAACATATGACCG
*frk3–1* (SALK_044085)	gDNA	CGTCCTATCTCCATCAAAGC
	gDNA/T-DNA	CGCTTGTCGTCGGTACAA
	cDNA flanking insert	GTTTTACCGAAACCCGAGTG
	cDNA flanking insert	CCGCCTTAGCTGCAGAAATA
*frk3–2* (SALK_035386)	gDNA	CCGCCTTAGCTGCAGAAATA
	gDNA/T-DNA	GTTTTACCGAAACCCGAGTG
	cDNA flanking insert	GTTTTACCGAAACCCGAGTG
	cDNA flanking insert	CCGCCTTAGCTGCAGAAATA
*frk5–1* (SALK_027635)	gDNA	CGCTTTTGTCACTTTGCGTTCTGAC
*frk5–2* (SALK_057002)	gDNA/T-DNA	CGAGTACGGACTGGTCATCAACAA
	cDNA flanking insert	GATGATTTCGGTCATATGCTCGC
	cDNA flanking insert	GGAGAAGCCGTACAACACCA
*frk7–1* (GABI_253H07)	gDNA	CAGCACGGTCATCAACAATC
	gDNA/T-DNA	TAGCGAACGGTGCTACATCA
	T-DNA (GABI)	ATATTGACCATCATACTCATTGC
	cDNA flanking insert	AACGGTGTCTCTGCTGAAGG
	cDNA flanking insert	TCAGGAGGCTCTGAACTTCA
SALK T-DNA Left border	T-DNA in SALK lines	TGGTTCACGTAGTGGGCCATCG
Positive control gene (At1g49350)	cDNA	GCGTGGATGCCGTTGAAAAT

### Preparation of recombinant proteins

Proteins were expressed in *E. coli* strain BL21-pLys-S (New England Biolabs) and purified via Ni Sepharose (GE Healthcare) affinity chromatography. Bacterial cells were lysed by sonication in lysis buffer (50 mM Tris, 100 mM NaCl, 50 mM imidazole, pH 7.5, complete mini protease inhibitor cocktail (Roche)), mixed with resin and the beads washed with at least 10 column volumes of wash buffer (50 mM Tris, 1.5 M NaCl, 50 mM imidazole, pH 7.5) and eluted in elution buffer (50 mM Tris, 100 mM NaCl, 300 mM imidazole, pH 7.5). Eluate was brought to 20% glycerol and was flash frozen and stored at −80 °C. Concentration of recombinant proteins was determined using Protein Assay Reagent (BioRad) and BSA to generate standard curves. Purity was determined by coomassie staining of an 10% SDS-acrylamide gel.

### Enzymatic assays

Enzymatic assays were carried out in assays coupling the production of ADP to NADH oxidation and monitored by A_340_. The reaction mixture consisted of 50 mM Tris pH 7.5, 100 mM KCl, 10 mM MgCl_2_, 0.1 mM ATP, 1 mM phosphoenolpyruvate, 10 mM inorganic phosphate, 1 mM NADH, 1 mM fructose, 2 U/mL lactate dehydrogenase, 2 U/mL pyruvate kinase. Reactions were carried out in 100 μl volumes in microplate format (NUNC) and A_340_ was monitored using a Spectra Max 340 microplate reader (Molecular Devices). For the determination of Km values of ribose and ATP, initial reaction rates were measured in concentrations of ribose or ATP, respectively, varied between 2 and 0.0625 mM with all other parameters held constant. Data were plotted and biochemical parameters determined via non-linear curve fitting to either the Michaelis Menten equation or substrate inhibition equation as appropriate in Prism 7 (GraphPad). To determine the effect of monovalent cations, initial reaction rates were determined in the presence of 100 mM KCl, or NaCl and normalized to that of KCl. To determine the effect of divalent cations, initial reaction rates were determined in the presence of 10 mM MgCl_2_ or EDTA, and normalized to that of MgCl_2_. Inorganic phosphate was omitted from the reaction to test its requirement. Nucleotide preference was tested by substituting the 0.1 mM ATP with 0.1 mM GTP or UTP, and normalized to that of 0.1 mM ATP. All assays to determine Vmax and Km were performed in triplicate. Assays to determine cofactor requirements were performed in quadruplicate and expressed as scatterplots showing all data points. Statistical differences were determined using two-way analysis of variance and post hoc Dunnett’s multiple comparison test in Prism (GraphPad).

### Microscopy

Agrobacteria strain AGL1 (gift from Charles Gasser, Department of Molecular and Cellular Biology, UC-Davis [48]) carrying vectors to express C-terminally YFP-tagged FRKs were co-infiltrated with AGL Agrobacteria carrying P19 (gift from Richard Michelmore, Plant Sciences Department, UC-Davis [49]) into *Nicotiana benthamiana* leaves. After 48 h, a small leaf disc close to the site of infiltration was excised and then prepared and mounted similarly to what was shown in Littlejohn and Love [50]. Briefly, leaf discs were soaked in perfluorodecalin for 5 min and then mounted on slides in a small well created with Carolina Observation Gel (Carolina Biological Supply Company), covered with a coverslip, and imaged on an Olympus FV1000 confocal microscope using a 40× objective and the appropriate filters.

## Abbreviations

ADK: Adenosine kinase
Col-0: Columbia ecotype Arabidopsis
cTP: Chloroplast transit peptide
FRK: Fructokinase
PFK-2: *E. coli* phosphofructokinase-2
pfkB: Phosphofructokinase B
YFP: Yellow fluorescent protein
